# Assessing factors associated with nurses leaving the profession: A secondary analysis of cross-sectional data

**DOI:** 10.1016/j.ijnsa.2025.100315

**Published:** 2025-02-27

**Authors:** Björn Lantz, Carina Fagefors

**Affiliations:** a Technology Management and Economics, Chalmers University of Technology, Gothenburg, Sweden; b Sahlgrenska University Hospital, Gothenburg, Sweden

**Keywords:** Nurse retention, Nurse staff, Nurse turnover, Sweden

## Abstract

**Background:**

Nurse turnover is a global challenge affecting workforce stability, financial sustainability of healthcare systems, and the quality of care. Despite extensive research, a limited number of researchers have explored the actual reasons behind nurses leaving the profession as opposed to leaving their job, particularly in relation to demographic variables.

**Objective:**

We aimed to explore how demographic factors—age, sex, and work area—influenced nurses' experiences of key turnover factors, providing insights to inform tailored retention strategies.

**Design:**

Secondary cross-sectional analysis of national survey data.

**Setting(s):**

Data were collected by Statistics Sweden through a mail survey.

**Participants:**

The sample consisted of 2,860 individuals with nursing qualifications who had left the profession between 2005 and 2013 and responded to a 2016 survey.

**Methods:**

The analysis involved exploratory factor analysis and structural equation modeling to identify and examine five latent factors contributing to nurses leaving the profession. The researchers assessed the impact of demographic variables on these factors.

**Results:**

Five primary factors were identified: compensation and fairness, psychosocial work environment, career development constraints, non-clinical workload, and support and safety. Younger nurses and those outside primary care reported greater dissatisfaction across multiple factors, while female nurses faced heightened psychosocial strain. Work area differences also influenced perceptions of support and workload.

**Conclusions:**

We have underscored the significant role of demographic variables associated with leaving the nursing profession. Tailored interventions, such as structured mentorship for younger nurses, family-supportive policies for female nurses, and workload optimization in high-stress areas, may be helpful. The results are limited by the age of the data, which predates recent healthcare shifts, emphasizing the importance of regular workforce monitoring.

**Registration:**

Not registered

**What is already known about the topic**

• Nurse turnover has been widely studied, with common factors including work-related stress, dissatisfaction with compensation, and limited career development opportunities contributing to workforce instability.
• Previous researchers have highlighted that demographic characteristics, such as age, sex, and work area, can shape nurses’ perceptions of work environment challenges and contribute to turnover.

Alt-text: Unlabelled box

**What this paper adds**

• We identified five categories of reasons nurses gave for leaving the profession, with demographic factors, such as age and work area, influencing perceptions of compensation, leadership support, and career development opportunities.
• Younger nurses and those in non-primary care settings expressed greater dissatisfaction across several categories, highlighting the possible need for demographic-specific retention strategies.
• By analyzing a sample of nurses who had already left the profession, rather than those stating an intention to leave, we have contributed to the validity and reliability of findings in previous research.

Alt-text: Unlabelled box

## 1 Background

Nurses leaving their jobs is a significant global issue with far-reaching implications for healthcare systems, particularly in terms of quality of care and financial sustainability of healthcare systems (Brook et al., 2019). In Sweden, the nurse turnover rate was reported at 13.9 % in 2023 (SKR, 2024), reflecting broader trends seen internationally. In a recent meta-analysis, Ren et al. (2024) found that the global nurse turnover rate (including intention to leave) ranged between 8 % and 36.6 %, with an average of 16 %. This places Sweden's rate close to the global average, underscoring the universal challenge nurse turnover presents to healthcare systems. High turnover rates place immense strain on healthcare systems, leading to staff shortages, increased recruitment costs, and diminished patient outcomes (Brook et al., 2019).

Turnover in the nursing profession can be divided into two types: organizational turnover, where nurses move between employers, and professional turnover, where nurses leave the profession entirely (Halter et al., 2017). The latter presents a critical challenge for healthcare, particularly given the growing demand for specialized nursing skills and the slow replacement of experienced professionals. Despite a wealth of research on nurses’ intention to leave, relatively few studies have focused on the actual reasons for nurses permanently exiting the profession. Although intention to leave might be correlated with actual turnover, methodological issues can arise, as stated intentions are not always followed by actions (Bolt et al., 2022; Cho and Lewis, 2012). In implementing effective nurse retention policies, a deep understanding of the reasons behind actual turnover has practical and academic implications (Halter et al., 2017; Bolt et al., 2022). Understanding these reasons is essential for developing effective retention strategies (Brook et al., 2019).

In Sweden, the shortage of nurses in primary healthcare has become particularly alarming in recent years. Karlsson et al. (2024) highlighted that 21.1 % of Swedish primary healthcare nurses expressed dissatisfaction with their jobs, citing poor leadership, insufficient social support, and change fatigue as major contributing factors. This dissatisfaction was closely linked to high turnover intentions, which, if realized, could further aggravate the existing nursing shortage in Sweden's primary care sector—a sector responsible for a significant portion of healthcare services. Primary healthcare in Sweden, as in many other countries, is expected to take on an even greater role in the future as part of efforts to develop more integrated and holistic care models aimed at preventing disease and managing chronic conditions (Karlsson et al., 2024). Therefore, addressing the factors driving nurses to leave the profession in this sector is crucial for maintaining a stable and effective healthcare workforce.

Researchers have consistently highlighted several key drivers of both types of nurse turnover, including work-related stress, limited career advancement opportunities, and insufficient compensation (Brook et al., 2019). Swedish nurses responded in a recent survey that discontent with their current job was significantly associated with poor leadership climate, lack of social support from colleagues, fatigue, poor health, and working >40 h per week (Karlsson et al., 2024). These findings align with international researchers, such as Ejebu et al. (2024), who highlighted both common and career-stage-specific "push-pull" factors influencing nurses' decisions to leave or remain in the profession.

Additionally, compensation and workload imbalances, particularly in relation to the responsibilities and education required for nursing roles, remain significant sources of dissatisfaction (Brook et al., 2019). Importantly, factors influencing turnover often vary across demographic groups, with younger nurses and those in non-primary care settings experiencing higher dissatisfaction. Karlsson et al. (2024) emphasized that addressing these issues requires targeted interventions to enhance job satisfaction and reduce organizational turnover in Sweden's primary healthcare system.

Researchers studying nurse retention have demonstrated the effectiveness of targeted interventions, such as improved leadership support, better career development opportunities, mentoring, residency, and transition programs, in addressing career development, work-life balance, and job satisfaction. For example, Brook et al. (2019) reported that such programs have significantly reduced early-career nurse turnover when implemented effectively. Many researchers have concentrated on intent to leave within organizational contexts, offering valuable insights into workforce retention strategies. For example, Cao et al. (2021) and Enea et al. (2024) investigated turnover intentions among nurses in China and Europe, respectively, identifying key predictors, such as sociodemographic factors, burnout, and job dissatisfaction. Bell and Sheridan (2020) analyzed intention to stay, focusing on the role of organizational commitment and its relationship with burnout and job satisfaction. Other researchers have explored novel factors, including the influence of illegitimate tasks on work motivation and burnout, as shown in studies conducted in Sweden (Ahlstedt et al., 2023) and Ecuador (Moncayo-Rizzo et al., 2024). Despite these contributions, gaps remain in understanding professional turnover, where nurses leave the profession entirely. Ejebu et al. (2024) addressed this gap by emphasizing the need for strategies tailored to prevent nurses from exiting the profession, with a focus on long-term career growth and retention across different career stages.

In this study, we aimed to explore the demographic and professional factors associated with nurses leaving the profession and to provide insights for developing more tailored retention strategies. Following this introduction, Section 2 presents a literature review focusing on how demographic variables—age, sex, and work area—shape nurses' experiences of turnover factors. Section 3 outlines the methodological considerations and analytical approaches used. Section 4 presents the study's findings. Section 5 discusses the results in relation to practice and previous research, and presents recommendations for targeted retention strategies as well as directions for future research. Section 6 concludes the paper.

## 2 Literature review

An improved understanding of the reasons associated with professional turnover is essential to addressing the global nursing shortage and its impact on healthcare systems. Unlike organizational turnover, which involves switching employers, professional turnover reflects deeper systemic and individual challenges that remain underexplored. An exception is Chênevert et al. (2016), who identified unmet expectations, diminished professional self-image, and insufficient support systems as key predictors of professional turnover.

By highlighting both gaps in the literature and persistent patterns across contexts, we have aimed to provide an overview of the dynamics associated with professional exits in nursing. Understanding how demographic variables, such as age, sex, and work area, could influence nurses' reasons for leaving the profession may provide critical insights for designing effective retention strategies tailored to the diverse needs of the nursing workforce (e.g., Bao et al., 2023; Enea et al., 2024). These variables are relatively stable and are associated with individual experiences within the workforce, making them relevant factors in understanding work-related challenges. While we could not directly establish the identified factors (e.g., compensation and fairness, psychosocial work environment, and career development constraints) as causal antecedents of turnover, evidence strongly links such factors to turnover intentions.

### 2.1 Age and career stage challenges

Age seems to influence professional turnover differently across career stages. Brook et al. (2019) explored how younger nurses experience "transition shock," where the realities of nursing practice fail to meet pre-entry expectations. Mihailovic et al. (2022) reported that stress, inadequate mentorship, and feelings of incompetence frequently diminish professional self-image, prompting some nurses to consider leaving the profession. Researchers from Sweden showed that one in five newly graduated nurses reported intentions to leave the profession within 3 years, largely due to these challenges (Mihailovic et al., 2022).

For older nurses, barriers such as physical strain, career stagnation, and limited opportunities for professional advancement can lead to decisions to leave the profession. Twigg and McCullough (2014) observed that while older nurses often demonstrate resilience through strong professional networks, systemic barriers such as stagnation can contribute to professional exits, particularly as retirement approaches.

### 2.2 Sex-based differences in professional turnover

Sex-based differences also seem to shape patterns of professional turnover. Bao et al. (2023) and Yun et al. (2021) examined how family-work conflicts, rigid work schedules, and insufficient family-supportive policies disproportionately affect female nurses, possibly leading some to exit the profession. These structural challenges highlighted significant inequities within the profession.

Male nurses face distinct challenges due to their minority status in the workforce. Cowin et al. (2008) documented how male nurses often experience a lack of relatedness in predominantly female workplaces, which undermines job satisfaction and long-term professional commitment. Mihailovic et al. (2022) also associated societal stereotypes about nursing as a female-dominated profession with decisions by some men to leave the field.

### 2.3 Work area and professional retention

The characteristics of a nurse's work area can also influence professional turnover. Kerzman et al. (2020) and Sasso et al. (2019) examined high-stress environments such as intensive care units, emergency rooms, and neonatal units, finding that chronic emotional strain, heavy workloads, and high patient acuity contributed to burnout, which can lead to professional exits. Ren et al. (2024) emphasized that unresolved challenges in these settings are associated with decreased professional commitment, which may contribute to some nurses considering alternative careers.

In contrast, Twigg and McCullough (2014) discussed how nurses in outpatient or administrative roles experience lower professional turnover rates, which they attributed to more predictable work conditions and reduced emotional labor. These researchers suggested that addressing the specific stressors of different work environments could enhance professional retention.

### 2.4 Theoretical frameworks

The Push-Pull Theory and Self-Determination Theory offer useful frameworks for understanding professional turnover. Sasso et al. (2019) and Ren et al. (2024) described how push factors, such as heavy workloads, burnout, and lack of professional development opportunities, may drive nurses away from the profession. High-stress environments, particularly in intensive care units or emergency rooms, exacerbate these factors (Kerzman et al., 2020). Pull factors, such as opportunities for better work-life balance, higher salaries, or less stressful work conditions, draw nurses toward alternative careers. Bao et al. (2023) noted that the absence of family-friendly policies or career growth opportunities makes such alternatives more appealing, particularly for female nurses balancing caregiving responsibilities.

The intrinsic psychological needs of autonomy, competence, and relatedness, as emphasized by the Self-Determination Theory, are central to understanding professional turnover (Conroy et al., 2023; Yun et al., 2021). Brook et al. (2019) found that inadequate support systems or insufficient feedback often undermine competence, particularly among early-career nurses. Mihailovic et al. (2022) observed that the absence of meaningful workplace relationships weakens relatedness, contributing to disengagement and decisions to leave the profession.

Leadership influences professional turnover indirectly by shaping environments that support career satisfaction and professional identity. Conroy et al. (2023) described how transformational leadership, which aligns closely with the Self-Determination Theory, fosters autonomy, competence, and relatedness by promoting mentorship programs and professional development opportunities. Brook et al. (2019) identified such programs as particularly valuable for younger nurses navigating early-career challenges.

For older nurses, transformational leadership that prioritizes autonomy and recognizes expertise reinforced professional commitment (Yun et al., 2021; Mihailovic et al., 2022). Although leadership interventions often focus on organizational retention, they may also contribute to reducing professional exits by addressing systemic and individual challenges.

### 2.5 Culturally-specific contexts

Researchers conducting studies in culturally-specific contexts, such as Sweden, remain limited in number (e.g., Mihailovic et al., 2022; Ahlstedt et al., 2023; Karlsson et al., 2024). Swedish nurses face unique challenges, including high expectations for equity and work-life balance, which are underrepresented in global studies (Mihailovic et al., 2022; Cao et al., 2021). Additionally, the impact of emerging stressors, such as illegitimate tasks—work perceived as unnecessary or misaligned with professional roles—is not fully understood. These tasks significantly contribute to burnout and turnover, particularly in high-stress environments (Moncayo-Rizzo et al., 2024; Yun et al., 2021).

## 3 Methods

We followed a cross-sectional design using secondary data from a survey conducted by Statistics Sweden in 2016 (Holm and Morell, 2017). We reported the results in accordance with the STROBE guidelines (Strengthening the Reporting of Observational Studies in Epidemiology) to ensure quality and transparency. The original report by Holm & Morell (2017) has been published only in Swedish with a summary in English. The aim was to explore factors contributing to professional turnover among nurses who had left the profession.

The data were drawn from a survey of individuals with nursing qualifications in Sweden who had left the nursing profession between 2005 and 2013. A total of 4206 individuals were contacted, and 2860 responded, resulting in a response rate of 68 %. Participants were selected from the Swedish Occupational Register and were eligible if they had worked as nurses between 2005 and 2013 but left the profession in 2013 or earlier. Individuals who had returned to clinical work by 2016 were excluded. The final sample was representative of the Swedish nursing population in terms of sex, age, and work area, and nurses with foreign degrees were excluded to maintain consistency in the dataset. The majority of respondents (84.9 %) were female, and more than half had worked in primary care (the first level of healthcare, providing general medical services for prevention, diagnosis, and treatment of common health issues, often delivered by general practitioners or family doctors) before leaving the profession. The average age of respondents was 44.7 years, with an average of 13 years of experience in the nursing profession. The participants' ages ranged from 25 to 65, and their years of experience (not considered in this study) ranged from 0 to 48. Further details regarding sampling and data collection can be found in the original report by Holm & Morell (2017).

Data were collected using a structured questionnaire developed by Statistics Sweden. The questionnaire contained 50 items, 15 of which specifically focused on reasons for leaving the nursing profession. These 15 items were rated on a 4-point Likert-type scale ranging from "contributed fully" (step 1) to "contributed nothing" (step 4). These 15 items are listed in Table 1. The questionnaire was developed as part of a national initiative by Statistics Sweden to explore workforce issues among nurses and was based on a review of previous research in the field. However, no specific psychometric testing or validation data were provided in the original report by Holm & Morell (2017).

**Table 1:** Descriptive statistics.

Item	*n*	Mean	S.D
Q12. Lack of safety due to threats or violence from patients or relatives	2039	3.79	0.56
Q1. Too many contacts with patients	2096	3.60	0.78
Q15. Payment in relation to other nurses in the workplace	2024	3.50	0.86
Q7. Too physically demanding	2103	3.49	0.84
Q2. Too much administrative work	2117	3.41	0.84
Q11. Lack of support in difficult situations with patients or relatives	2096	3.33	0.95
Q3. Too many non-nursing-related tasks	2092	3.32	0.87
Q5. Lack of opportunities for competence development	2114	2.82	1.09
Q6. Lack of career options	2095	2.80	1.15
Q8. Too mentally demanding	2147	2.73	1.12
Q10. Bad leadership	2116	2.66	1.13
Q4. Too heavy workload in relation to the working time	2137	2.54	1.12
Q13. Payment in relation to the workload	2132	2.46	1.11
Q14. Payment in relation to education	2115	2.41	1.11
Q9. Too low job control	2155	2.28	1.07

Note. *Q*= item number in questionnaire, *n*= sample size, S.D.= standard deviation.

In addition to the turnover-related items, the analysis also incorporated three demographic variables as independent variables (IVs): sex, age, and area of work.

• SEX: Sex was coded as a binary variable (0 = female, 1 = male), as the study aimed to explore potential differences between male and female nurses regarding their reasons for leaving the profession.
• AGE: Age was included as a continuous variable representing the respondent's age upon leaving the profession.
• AREA: Area of work was coded as a binary variable, distinguishing between nurses who worked in primary care (coded as 0) and those in other healthcare settings (coded as 1).

These variables were selected based on existing literature, which suggests that demographic factors, such as sex, age, and area of work, are associated with leaving the nursing profession.

We analyzed these data in two stages. In the first stage, exploratory factor analysis (EFA) was performed to identify the underlying dimensions of nurse turnover based on the 15 items related to reasons for leaving the profession. EFA is a statistical method that reduces a large number of observed variables into a smaller set of latent factors, each representing a distinct construct. Parallel analysis was used to determine the appropriate number of factors to retain, and Varimax rotation was applied to enhance the interpretability of the factor structure. Items that did not load clearly onto a factor or that loaded onto multiple factors were excluded to improve model clarity. This analysis allowed the items to be grouped into a few distinct factors that represent the core reasons for turnover.

Following the EFA, structural equation modeling (SEM) was employed to assess the relationships between the identified latent factors and the demographic variables. SEM was chosen because it allows for the simultaneous examination of multiple relationships between observed and latent variables, providing a comprehensive understanding of how demographic factors influence turnover behavior. Standard fit indices, such as the root mean square error of approximation (RMSEA), the comparative fit index (CFI), and the Tucker-Lewis index (TLI), were used to assess the model's overall fit. Thus, while we employed a data-driven approach to uncover underlying factors, the findings provided a basis for hypothesis-driven research, thus contributing to the development of a theoretical framework for understanding nurses’ decisions to leave the profession. Jamovi (v. 2.3.28) was used for all data analyses.

The internal consistency of the identified factors was evaluated using Cronbach's alpha and composite reliability (CR) measures. These tests ensured that the items within each factor reliably measured the same underlying construct. Additionally, discriminant validity was assessed by comparing the average variance extracted (AVE) values for each factor to the squared inter-factor correlations. This confirmed that the identified factors were distinct and measured different aspects of nurse turnover.

The data collection by Statistics Sweden was conducted following national ethical guidelines for research involving human participants. Participation in the survey was voluntary, and respondents were informed of the purpose of the study and their rights. Consent was implied through completion and return of the survey. All data were anonymized by Statistics Sweden before analysis, and no identifiable personal information was included in the dataset made available to the researchers. The institutional ethical advisory board at our university reviewed our study and determined that no further ethical review was required beyond the procedures conducted by Statistics Sweden.

## 4 Results

This section begins with descriptive statistics for the 15 items related to reasons for nurse turnover, providing an overview of the key factors that respondents identified as influencing their decision to leave the profession. Following this, we applied EFA to uncover the underlying dimensions contributing to turnover. After refining the model by removing two items, five distinct factors were identified. Finally, we employed SEM to examine how these factors were influenced by demographic variables, such as sex, age, and area of work. The combined findings offered a comprehensive view of the drivers behind nurse turnover and the demographic patterns associated with these factors.

Table 1 displays the 15 items related to the reasons for leaving the nursing profession and their descriptive statistics. From the descriptive perspective, the items “Too many contacts with patients” and “Lack of safety due to threats or violence from patients or relatives” contributed the least to the respondents’ leaving the profession, while the items “Too low job control” and “Payment in relation to education” contributed the most.

The analysis of these data using EFA identified five distinct factors contributing to nurses leaving the profession in Sweden. Initially, 15 items related to reasons for leaving the profession were analyzed, and after removing two items (Q1 and Q7) due to low factor loadings, the model improved significantly (Kline, 2016). As shown in Table 2, the five factors identified were Compensation and Fairness, Psychosocial Work Environment, Career Development Constraints, Non-Clinical Workload, and Support and Safety. Compensation and Fairness captured the largest variance, followed by Psychosocial Work Environment, highlighting that compensation-related concerns were a primary driver of turnover. The five factors explained 64.65 % of the total variance.

**Table 2:** Factor structure and reliability measures.

Item	Compensation and fairness	Psychosocial work environment	Career development constraints	Non-clinical workload	Support and safety
Q2				0.615	
Q3				0.953	
Q4		0.834			
Q5			0.857		
Q6			0.889		
Q8		0.873			
Q9		0.495			
Q10					0.435
Q11					0.833
Q12					0.450
Q13	0.840				
Q14	0.976				
Q15	0.378				
					
α	0.78	0.84	0.88	0.79	0.62
CR	0.85	0.86	0.88	0.79	0.68

Note. α= Cronbach's alpha. CR= composite reliability.

Compensation and Fairness captured dissatisfaction related to payment in relation to education, workload, and comparisons with other nurses. Psychosocial Work Environment reflected mental and emotional strain in the workplace, while Career Development Constraints pointed to limited opportunities for professional growth. Non-Clinical Workload highlighted dissatisfaction with administrative and non-nursing-related tasks, and Support and Safety emphasized concerns about workplace safety and lack of support.

The overall model fit indices supported the robustness of the factor structure. The RMSEA was 0.063 (90 % CI: 0.054 to 0.071), the TLI was 0.951, and the CFI was 0.902, indicating that the five-factor model was a good fit for the data (Hair et al., 2010). Bartlett's test of sphericity was statistically significant (χ²= 11,452, df= 78, *p* < 0.001), and the Kaiser-Meyer-Olkin measure of sampling adequacy was 0.799, confirming that the dataset was appropriate for factor analysis (Hair et al., 2010).

Reliability was measured through Cronbach's alpha and composite reliability (see Table 2). Most constructs demonstrated good internal consistency, with Cronbach's alpha values above 0.70. Support and Safety had a slightly lower value (0.62), but it remained within an acceptable range for exploratory research. The composite reliability values supported these findings, confirming the reliability of the measurement model (Hair et al., 2010).

Discriminant validity ensures that the factors identified are distinct from each other, meaning that each factor captures a unique aspect of nurse turnover. In this study, discriminant validity was confirmed by ensuring that the AVE values for each factor were greater than the squared inter-factor correlations (see Table 3), indicating that the constructs were distinct (Fornell and Larcker, 1981). Note that all inter-factor correlations were statistically significant (*p* < 0.001) due to the large sample size.

**Table 3:** Inter-factor correlations and average variance extracted.

	Compensation and fairness	Psychosocial work environment	Career development constraints	Non-clinical workload	Support and safety	AVE
Compensation and fairness	–	0.3933	0.4518	0.2765	0.2699	0.68
Psychosocial work environment		–	0.1263	0.4998	0.5662	0.67
Career development constraints			–	0.1756	0.2279	0.78
Non-clinical workload				–	0.4033	0.65
Support and safety					–	0.44

Note. AVE= average variance extracted.

Convergent validity was assessed by calculating the AVE for each factor, and most factors met the recommended threshold of 0.5 (Hair et al., 2010). Compensation and Fairness, Psychosocial Work Environment, Career Development Constraints, and Non-Clinical Workload showed strong convergent validity, while Support and Safety had a slightly lower AVE yet still demonstrated meaningful construct validity (see Table 3).

The SEM analysis was conducted to explore the relationships between the identified factors contributing to professional turnover among nurses and the demographic variables. The analysis revealed a number of significant associations between these variables and the factors contributing to nurse turnover (see Table 4).

**Table 4:** Results from the SEM analysis.

*Dependent variable*	*Predictor*	*β*	*z*	*p*
Compensation and fairness	SEX	-0.006	-0.23	0.817
	AGE	0.167	6.33	< .001
	AREA	-0.065	-2.60	0.009
Psychosocial work environment	SEX	0.150	6.07	< .001
	AGE	0.230	9.24	< .001
	AREA	0.007	0.28	0.782
Career development constraints	SEX	-0.024	-0.95	0.342
	AGE	0.091	3.61	< .001
	AREA	-0.065	-2.60	0.009
Non-clinical workload	SEX	-0.026	-0.96	0.337
	AGE	0.036	1.36	0.174
	AREA	0.097	3.62	< .001
Support and safety	SEX	0.061	2.17	0.030
	AGE	0.141	4.91	< .001
	AREA	0.075	2.66	0.008

Note. SEM= structural equation modelling, *β*= path coefficient, *z* = *Z*-score, *p* = *p*-value.

The Compensation and Fairness factor was statistically significantly influenced by AGE and AREA. This indicates that younger nurses, as well as those working outside primary care, were more likely to report dissatisfaction related to compensation as a reason for leaving the profession. For the Psychosocial Work Environment factor, SEX and AGE were statistically significant predictors, with female and younger nurses reporting higher levels of dissatisfaction related to psychosocial aspects of their work. These findings suggest that emotional strain and lack of support disproportionately affect these demographic groups.

The Career Development Constraints factor showed a statistically significant negative relationship with AREA and AGE, indicating that younger nurses and nurses outside primary care felt more limited in their career advancement opportunities. The Non-Clinical Workload factor was also statistically significantly influenced by AREA, with nurses in primary care reporting higher dissatisfaction due to administrative and non-nursing tasks. Lastly, the Support and Safety factor was statistically significantly influenced by both SEX, AGE and AREA, with nurses in primary care, younger nurses, and female nurses more likely to express concerns about safety and lack of support in difficult situations.

In summary, from the analysis, we have highlighted that the demographic variables significantly influenced dissatisfaction in areas such as compensation, work environment, and safety concerns, offering critical insights into the complex dynamics associated with the reasons for nurses leaving the profession.

## 5 Discussion

In this study, we have demonstrated that demographic variables—age, sex, and work area—were critical factors associated with shaping nurses' perceptions of five turnover factors: compensation and fairness, psychosocial work environment, leadership and support, career development opportunities, and work-life balance. By examining these relationships, we have provided insights into enduring dynamics that may influence nurses to leave the profession and extend existing research on associated demographic factors.

Age was significantly associated with turnover drivers. Younger nurses were more likely to report dissatisfaction with compensation and fairness, as well as higher levels of stress and burnout. This aligns with previous findings on transition shock, where early-career nurses face challenges adjusting to the realities of professional practice (Brook et al., 2019; Mihailovic et al., 2022). Younger nurses frequently experience challenges such as insufficient mentorship, unclear career progression, and inequitable pay structures, which exacerbate dissatisfaction during the early stages of their careers (Calleja et al., 2024). Conversely, older nurses demonstrated greater resilience, likely reflecting accumulated professional experience and coping mechanisms. However, physical strain and limited advancement opportunities were common concerns among this group. Twigg and McCullough (2014) found that older nurses often valued flexible scheduling and reduced physical demands to address these challenges. Thus, it seems like the challenges faced by nurses at different career stages are influenced by stable developmental and career-related dynamics, which are unlikely to vary substantially over time or across contexts.

Sex also shaped factors associated with leaving the profession in significant ways. Female nurses disproportionately reported psychosocial strain and family-work conflicts, reflecting long-standing challenges in balancing professional and personal responsibilities. This is consistent with Yun et al. (2021) and Bao et al. (2023), who emphasized the negative impact of rigid work schedules and insufficient family-supportive policies on female nurses’ retention. Male nurses, although a smaller demographic group, faced unique challenges, including workplace isolation and role-related tension. These findings are similar to those of Cowin et al. (2008), who noted that societal expectations and workplace culture can undermine male nurses’ satisfaction and sense of belonging. While healthcare policies and practices may evolve, these dynamics are rooted in cultural and organizational structures, making them persistent over time.

Work area emerged as a possible determinant of turnover experiences, particularly in high-stress environments, such as intensive care units and emergency rooms (e.g., Sasso et al., 2019; Dunning et al., 2024). These environments are characterized by high patient acuity, emotional strain, and inadequate staffing levels, which consistently contribute to burnout and dissatisfaction (Sasso et al., 2019; Boulton and Farquharson, 2024). Conversely, outpatient and administrative roles were associated with fewer challenges, reflecting the importance of predictable work schedules and reduced emotional labor (e.g., Yun et al., 2021; Hernandez et al., 2024). The persistent nature of these work area dynamics reinforces the need for context-specific interventions. Rotational assignments from high-stress to lower-stress roles, additional staffing, and emotional support programs may help mitigate burnout and improve retention in high-acuity environments.

Demographic factors significantly shape turnover drivers in ways that are likely to exhibit stability over time and across healthcare systems. Age-related challenges like transition shock, sex-specific concerns (such as family-work conflicts), and work area stressors, such as burnout in intensive care units, reflect enduring dynamics that transcend contemporary healthcare changes. These patterns highlight the importance of developing targeted interventions that address the persistent challenges faced by different demographic groups in the nursing workforce.

We also contributed to theoretical frameworks by demonstrating the role of demographic variables in shaping the dynamics of the Push-Pull Theory and expanding the application of Self-Determination Theory in explaining nurse retention. The focus on autonomy, competence, and professional development as key elements in possibly reducing turnover highlights the value of Self-Determination Theory as a guiding framework for designing effective retention strategies.

We have highlighted the critical role of demographic variables—age, sex, and work area—in relation to nurses’ experiences of turnover factors. By identifying how these variables may influence perceptions of compensation, work-life balance, leadership, career development, and psychosocial environments, we may have provided actionable insights for addressing nurse retention challenges. For example, younger nurses may benefit from structured mentorship programs and transparent career progression pathways to mitigate dissatisfaction with compensation and fairness. Female nurses could benefit from family-supportive policies, such as flexible scheduling and childcare support, to reduce psychosocial strain and facilitate a better balance between professional and personal responsibilities. For nurses in high-stress environments, such as intensive care units, additional staffing resources, emotional support programs, and rotational assignments to lower-intensity roles may be critical for reducing burnout and improving retention. Leadership development programs that train managers to adopt transformational leadership styles could further address demographic-specific needs, such as mentoring early-career nurses and recognizing the contributions of late-career staff.

This study has several limitations. While the relationships between demographic variables and turnover factors were analyzed using SEM, the five factors were not tested as antecedents of actual turnover. This is due to the fact that all respondents in the dataset had already left the profession at the time of the survey in 2016, meaning the dataset could not capture turnover as an outcome. Additionally, the data analyzed predates recent healthcare shifts, such as those introduced by the COVID-19 pandemic, which may have amplified certain turnover drivers. Finally, the focus on the Swedish context may limit the generalizability of the findings, although Sweden's emphasis on equity may also offer insights for other similar healthcare systems.

Future researchers should validate these findings across diverse cultural and organizational contexts to ensure broader applicability. In addition, researchers should employ datasets that include both nurses who have left and those who have stayed, ideally in a longitudinal design, to explore how turnover factors directly influence turnover behaviour. They should also compare the intention to leave with actual leaving.

## 6 Conclusion

We have contributed to the understanding of the factors associated with nurses' decisions to leave the profession by highlighting the roles of age, sex, and work area. By identifying specific challenges associated with demographic factors, we may have provided actionable insights into developing tailored retention strategies. By emphasizing demographic-specific approaches, we have offered a basis for possible practical solutions to improve retention and reduce nurse attrition. Addressing these issues is essential for strengthening healthcare systems globally, particularly in the face of increasing demands on the nursing workforce. These insights may contribute to ongoing efforts to ensure the sustainability and resilience of healthcare systems worldwide.

## Declaration of generative AI and AI-assisted technologies in the writing process

During the preparation of this work the authors used ChatGPT 4o in order to copy edit the text. After using this tool/service, the authors reviewed and edited the content as needed and take full responsibility for the content of the publication.

## CRediT authorship contribution statement

**Björn Lantz:** Writing – review & editing, Writing – original draft, Methodology, Formal analysis, Data curation, Conceptualization. **Carina Fagefors:** Writing – review & editing, Conceptualization.

## Declaration of competing interest

None declared.
